# circIFITM1/miR-802/Foxp1 Axis Participates in Proliferation and Invasion of Lovo Cells

**DOI:** 10.1155/2022/7366337

**Published:** 2022-06-24

**Authors:** Yu Wan, Jinhua He, Zeping Han, Shengbo Wang, Yingying Zhao, Di Zeng, Ying Tang, Yiwei He, Lishan Zeng

**Affiliations:** ^1^Department of Gastroenterology, Panyu Central Hospital, Guangzhou, China; ^2^Department of Clinical Laboratory, Panyu Central Hospital, Guangzhou, China

## Abstract

**Objective:**

To explore the role of circIFITM1 and its potential molecular mechanism in colon cancer.

**Methods:**

The circIFITM1 in human samples and cell lines of colon cancer was measured via RT-PCR. The cyclicity of circIFITM1 was confirmed by agarose gel electrophoresis and Sanger sequencing, and the stability of circIFITM1 was confirmed by actinomycin D assay. The proliferative and invasive ability was detected by the CCK-8 assay and Transwell assay, respectively. RNA pull-down assay confirmed a combination of circIFITM1 and miRNA. Dual-luciferase reporter gene was used to detect the direct relationship between miRNA and the target gene.

**Results:**

circIFITM1 originated from the maternal gene IFITM1and had high stability. It was resistant to processing by actinomycin D. Upregulating circIFITM1 facilitated the proliferation and invasion of Lovo cells, while interfering with circIFITM1 expression inhibited them. circIFITM1 interacted with miR-802, and miR-802 targeted the 3′UTR of FOXP1. The overexpression of circIFITM1 downregulated miR-802 and upregulated FOXP1.

**Conclusion:**

circIFITM1 facilitates the proliferative and invasive abilities via miR-802/FOXP1 in Lovo cells.

## 1. Introduction

Recently, with the improved living standard and the changing lifestyle and diet structure, the development of colon cancer in China has grown over the years. Enhancing the earlier diagnosis and therapeutic rate is the primary means for a better prognosis of colorectal cancer patients [1]. However, the earlier diagnosis and therapy are very limited in advanced colon cancer patients for the fewer treatment options and poor outcome, resulting from the incompletely characterized development mechanism of colon cancer [2, 3].

As one of the endogenous noncoding RNA [4], circular RNA (circRNA) is expressed in mammals in a stable manner, has highly conserved sequences, and exerts an essential regulatory function on gene expression during the transcription or posttranscription processes through sponging microRNAs or acting as an RNA-binding protein and a nuclear transcription factor [5]. The anomalous circRNA expression is implicated in tumorigenesis and progression [6, 7].

hsa_circ_0020623 (circIFITM1) locates at chromosome 11 Chr11:313990-315272 and originates from the maternal gene interferon-induced transmembrane protein 1 (IFITM1). Our previous studies proved the overexpression of circIFITM1 in colorectal cancer, but the molecular mechanism was unclear. This study combined the results of bioinformatics analysis, using overexpression and blocking expression methods. To elucidate the molecular mechanism of circIFITM1 affecting the proliferative and invasive ability of colon cancer cells at the molecular level may provide a scientific basis for designing the strategy of inhibiting colon cancer metastasis targeting circIFITM1.

## 2. Materials and Methods

### 2.1. Bioinformatics

The online database (http://www.circbase.org/cgi-bin/simplesearch.cgi) was used to analyze the full-length sequence and localization of circIFITM1, and TargetScan was used to predict miRNA binding sites in circIFITM1.

### 2.2. Patient Information

The Central Hospital of Panyu District Archives offered clinical samples of colon cancer patients with the new diagnosis with patient informed consent and approval from the institutional Ethics Committee [8]. The basic patient information was shown in our previously published paper [8].

### 2.3. Cell Culture

Colo205 cells, DLD-1, HCT-116, HT-29, Lovo, RKO, SW480, SW620, Caco2, and HTC-8307 cells were obtained from Shanghai Cell Bank and cultured in the RPMI 1640 medium containing 10% newborn bovine serum at 37°C in a 5% CO_2_ incubator [9].

### 2.4. RT-PCR

RNA extracted using TRIzol reagent was reverse-transcribed following the instructions of GoScript™ Reverse Transcription System, whose product was applied for PCR as the template. The system and conditions of PCR were established based on the previous study [8]. The detailed PCR primers are presented in Table 1.

### 2.5. Agarose Gel Electrophoresis and Linkage Sequencing

2% agarose gel was prepared; then, when the temperature was about 55°C, l/20 Goodview nucleic acid dye was added and mixed well, the CDNA from PCR reaction was added to the Gel pore, the first pore was added to the DNA markers, and the selection voltage was 120 v, time set for 30 minutes. The precooled gel was put into the imaging analysis system to observe the size and position of the electrophoresis bands, and the amplified products were sent to Shanghai for sequencing.

### 2.6. Actinomycin D Assay

The colon cancer cell line was inoculated into a 6-well plate and was cultured overnight in low serum. Total RNA was extracted by 0, 12, and 24 h treatment with 2 g/l actinomycin D, respectively. After quantitative analysis, 500 ng was taken for reverse transcription and subsequent QPCR.

### 2.7. Cell Transfection and Grouping

siRNA sequence for circIFITM1 is 5′-GTGCACGTGCTTGTGAAAACA-3′; random sequence for negative control is 5′-GCAGAGCAGGTTCTGGGGCT-3′. All materials are purchased from Guangzhou Ruibo Biological Co., Ltd. When cell fusion degree was about 50%, the Lovo cells were transfected with 50 nmol/l si-circIFITM1 and random sequence according to the instruction of Lipofectamine 3000. The successfully transfected cells were digested and seeded, and four groups were assembled, including the blank control (Lipofectamine 3000 only), the negative control (random sequence), and siRNA (si-circIFITM1).

### 2.8. CCK-8-Assay

The grouping was the same to that of “1.7 cell transfection and grouping.” 10^3^ Lovo cells per well were seeded in 96-well plates with 100 *μ*l serum-free medium. At the transfection endpoint, every well was loaded with 10 *μ*l of CCK-8, accompanied by incubation for two hours and the measurement of absorbance (excitation wavelength 450 nm, reference wavelength 655 nm) with Bio-Rad. The cell proliferation inhibition rate was calculated: proliferation inhibition rate (%) = 100%A450 (control − experimental group)/A450 (control − blank group).

### 2.9. Transwell Assay

For the consolidation of Matrigel, 40 *μ*l of diluted Matrigel containing 30 *μ*l serum-free medium was loaded into the precooled Transwell cell before the incubation (37°C, two hours). Superfluous fluid was eliminated, and 100 *μ*l and 600 *μ*l serum-free media were loaded into the upper and the lower chamber, respectively, according to the previous study [10]. 10^5^ transfected cells with 100 *μ*l of the serum-free medium were loaded into the upper chamber and 600 *μ*l complete medium in the lower chamber. Following the incubation of 24 to 48 hours, cells in the upper chamber were entirely scrubbed. Cells on the upper and lower surfaces were stained with crystal violet and eluted with 33% acetic acid, accompanied by the measurement of OD570 using an enzyme-labeled instrument. The number of migrated cells was observed by a fluorescence microscopy.

### 2.10. RAP

Cell grouping is: (1) input group (positive control, biotin-free circIFITM1 probe, total RNA of lysis); (2) oligoprobe group (negative control, total RNA of lysis with random sequence); and (3) circIFITM1 probe group (RNA-complex captured by biotin-labeled circIFITM1 probe, 5′biotin-TTTCTCTTAAGTTTCTATTTCCTGCTGTTTTCACAAGCATCACAAGCACGTGCACTTTAT-3′). The constructed circIFITM1 vector was digested by Sal I, recycled, then transferred to the transcriptional mixture followed by the incubation (37°C, 2-5 hours) until the formation of cloudy liquid, and mixed with DNase I followed by the incubation (37°C, 15 min) [11]. Extracted RNA was biotin-tagged. To form a higher RNA structure, 3 *μ*g denatured RNA by heating at 90°C for 2 min was mixed with the same volume of RNA binding buffer before incubation (room temperature, 30 min). The mixture of folded RNA and 1 mg cell total protein was incubated (room temperature, 1 hour), then added with 40 *μ*l beads for the incubation at 4°C overnight. After washing eight times, magnetic beads were incubated with proteinase K (55°C, 30 min); then, the supernatant was shifted to another EP tube. Thereafter, RNA was extracted using phenol and chloroform, and miRNA was determined by qRT-PCR after reverse transcription [8].

### 2.11. Luciferase Reporter Assay

Cell grouping was performed [12], including control group (cell), NC group (scramble sequence+psiCHECK-2-circIFITM1-wt/psiCHECK-2- circIFITM1-mut; psiCHECK-2-FOXP1-3′UTR-WT/psiCHECK-2-FOXP1-3′UTR-mut), NCI group (inhibitor for scramble sequence+psiCHECK-2-circIFITM1-wt/psiCHECK-2- circIFITM1-mut; psiCHECK-2-IFITM1-3′UTR-WT/psiCHECK-2-IFITM1-3′UTR-mut), miR-802group (miR-802mimics+psiCHECK-2-circIFITM1-wt/psiCHECK-2-circIFITM1-mut; psiCHECK-2-FOXP1-3′UTR-WT/psiCHECK-2-FOXP1-3′UTR-mut), and miR-802 inhibitor group (miR-802 inhibitor+psiCHECK-2-circIFITM1-wt/psiCHECK-2-circIFITM1-mut; psiCHECK-2-FOXP1-3′UTR-WT/psiCHECK-2-FOXP1-3′UTR-mut). DNA of the Lovo cell was extracted for PCR amplification and acted as template for the amplification of the 3′UTR sequences of linear circIFITM1 and FOXP1 with the introduction of Xho and Not I restriction sites and protective bases. The amplified fragments of PCR products using restriction endonuclease of Xho and Notir were linked to psiCHECK-2 vector. The positive clones of transformed E. coli Dh5a were determined by restriction endonuclease, sequenced, and detected by double fluorescence [8]. The double luciferase activity test was performed strictly according to the product instruction provided by Promega.

### 2.12. Statistical Analysis

SPSS20.0 statistical software (IBM, USA) and GraphPad Prism 6.0 were used for data analysis. Measurements were expressed in standard deviation (SD). Parametric data were analyzed by one-way analysis of variance (ANOVA) and nonparametric data by the Kruskal-Wallis test, and a *P* < 0.05 was considered as statistically significant [13].

## 3. Results

### 3.1. circIFITM1 Was Derived from the Maternal Gene IFITM1. It Was Resistant to Actinomycin D Treatment and Had High Stability

**Figure 1:**
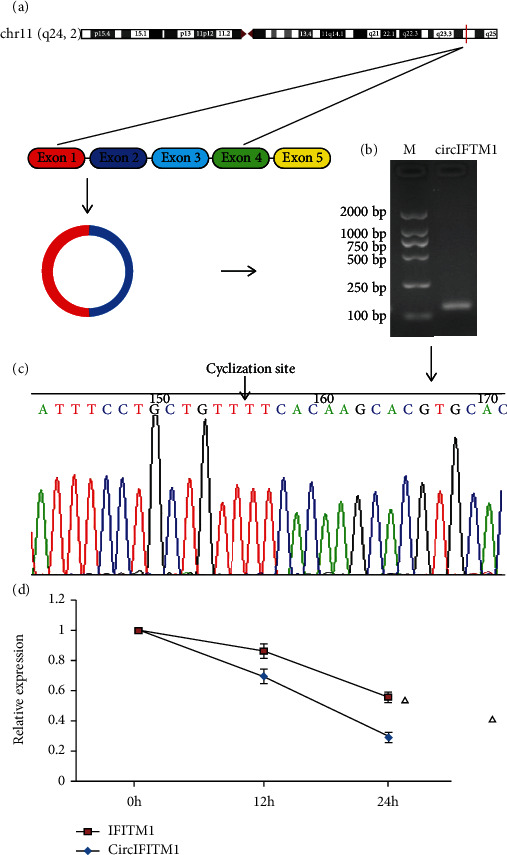
Characterization of cyclicity and stability of circIFITM1: (a) circIFITM1 schematic diagram; (b) identification of circIFITM1 cyclicity by agarose gel electrophoresis; (c) detection of circIFITM1 loop junction by Sanger sequence; (d) detection of circIFITM1 stability by actinomycin D assay, ^△^*P* < 0.05 compared with the IFITM1 group, *n* = 3.

circIFITM1 is located in chromosome 11 Chr11:313990-315272. circIFITM11 is derived from the maternal gene IFITM1 (interferon-induced transmembrane protein 1) and is exon 1 and exon 2 linked into a ring (Figure 1(a)). It was confirmed that the loop-forming site of circIFITM1 was located at T using agarose gel electrophoresis and Sanger junction sequencing (Figure 1(b)). Meanwhile, the level of circIFITM1was higher than that of linear IFITM1 after 12 and 24 hours of treatment with actinomycin D. The results showed that circIFITM1 was very stable (Figure 1(c)).

### 3.2. circIFITM1 Was Highly Expressed in Tissue and Cell Lines of Colon Cancer

**Figure 2:**
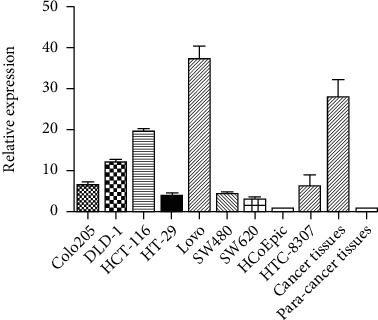
Detection of relative expression of circIFITM1 in colon cancer cell line and colon cancer tissue by RT-PCR assay.

The expressions of circIFITM1 in human colon cancer cell lines Colo205, DLD-1, HCT-116, HT-29, Lovo, RKO, SW480, SW620, and Caco2, HTC-8307 were determined through RT-PCR. The result displayed that Lovo cells expressed the highest level of circIFITM1, compared with HcoEPic cells. Moreover, the circIFITM1 expression in human colon cancer tissue was also higher than that in adjacent tissue (Figure 2).

### 3.3. circIFITM1 Impacted Proliferative and Invasive Abilities of Lovo Cells

**Figure 3:**
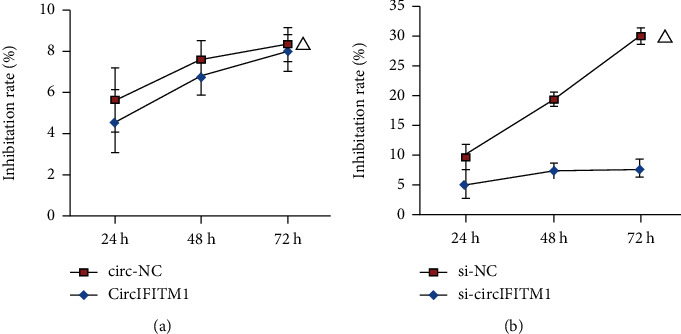
CircIFITM1 impacted the proliferation of Lovo cell line: (a) overexpression of circIFITM1; (b) knockdown of circIFITM1. Compared to the NC group: ^△^*P* < 0.05, *n* = 3.

**Figure 4:**
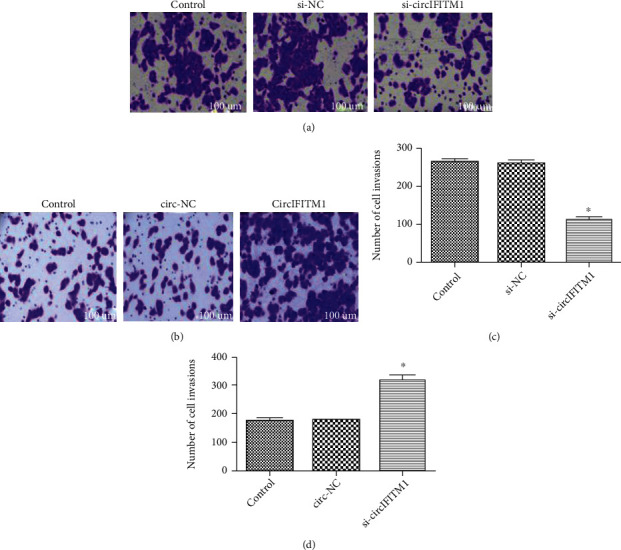
circIFITM1 impacted invasion of colon cancer cell line Lovo; (a, b) representative photographs of invasion (crystal violet staining, ×100), *n* = 3; (c, d) invasion number of Lovo cells. Compared to NC and control groups: ^∗^*P* < 0.05, *n* = 3.

After overexpressing and knocking down circIFITM1 in Lovo cells, si-circIFITM1 suppressed the proliferative and invasive abilities (Figures 3(a), 4(a) and 4(c)), while overexpression of circIFITM1 facilitated both abilities of Lovo cells (Figures 3(b), 4(b) and 4(d)). Those results demonstrated that circIFITM1 could promote proliferative and invasive abilities of Lovo cells.

### 3.4. circIFITM1 Interacts with miR-802, and miR-802 Targets 3′UTR of FOXP1

**Figure 5:**
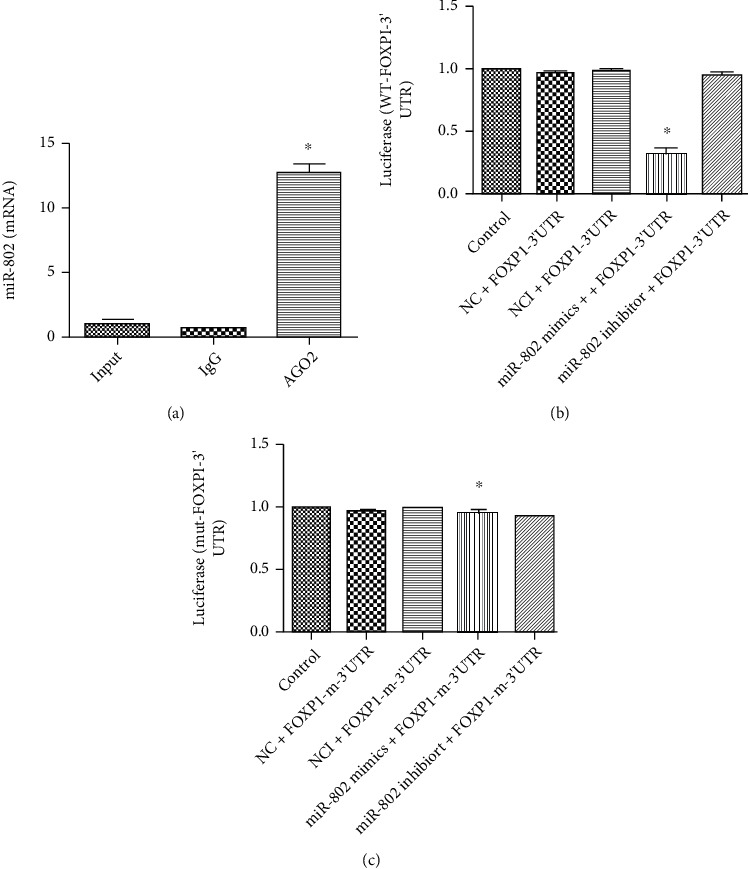
(a) Anti-AGO2 RIP Assay for exploring the relation of circIFITM1 and miR-802, ^∗^*P* < 0.05, compared to the input and IgG group. (b) Cotransfection of 293 T cells with miR-802 mimics and wild-type 3′UTR-FOXP1, ^∗^*P* < 0.05, compared to the control, NC, NCI, and miR-802 inhibitor group. (c) Cotransfection of 293T cells with miR-802 mimics and mutant 3′UTR-FOXP1, ^∗^*P* < 0.05, compared to control, NC, and NCI.

For the exploration of the molecular mechanism of circIFITM1 regulating, the interaction between circIFITM1 and miR-802 was detected, and it was found that the level of miR-802 in AGO_2_ antibody bound RNA was 13-folds greater than that of negative control (Figure 5(a)). In addition, further dual-luciferase reporter proved the direct binding of miR-802 and 3′UTR of FOXP1 (Figures 5(b) and 5(c)).

### 3.5. circIFITM1 Downregulated miR-802 and Upregulated FOXP1

**Figure 6:**
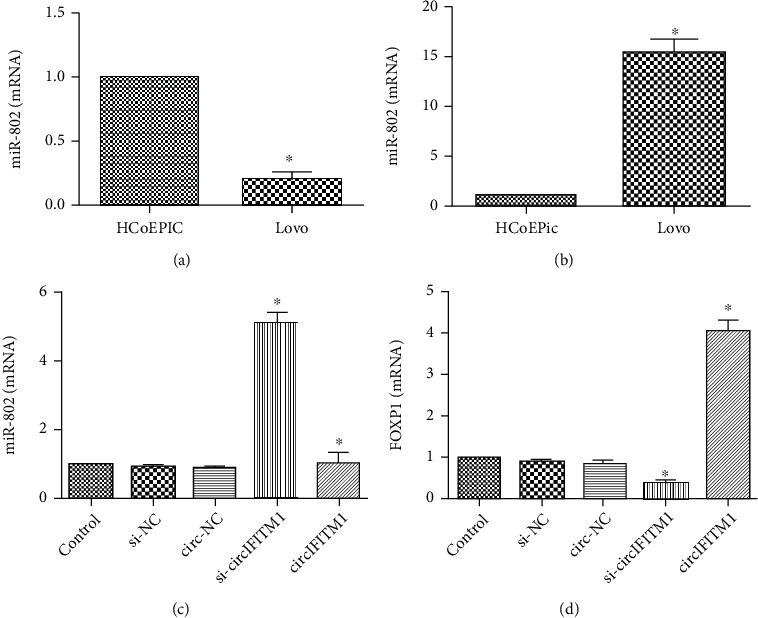
Connection of circIFITM1 to miR-802 and FOXP1: (a) miR-802 expression of Lovo cells detected by RT-PCR, ^∗^*P* < 0.05, compared to the HcoEPic cell group; (b) the FOXP1 expression of Lovo cells, ^∗^*P* < 0.05, compared to the HcoEPic cell group. (c, d) After increasing and decreasing the expression of circIFITM1 in Lovo cells, miR-802 and FOXP1 were examined using RT-PCR, respectively. Compared to the control and NC group: ^∗^*P* < 0.05 and ^∗∗^*P* < 0.05.

**Figure 7:**
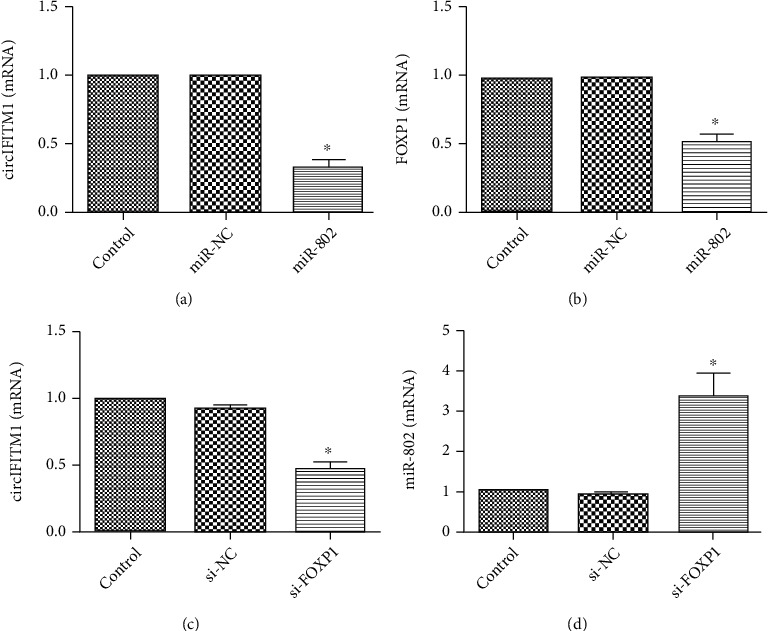
Relative gene expression detected by RT-PCR: (a) relative expression of circIFITM1 after transfection of the Lovo cell line with miR-802 mimic for 48 h; ^∗^*P* < 0.05, compared with the NC and control groups; (b) relative expression of FOXP1 after transfection of the Lovo cell line with miR-802 mimic for 48 h; ^∗^*P* < 0.05, compared with the miR-NC and control groups; (c) relative expression of circIFITM1 after transfection of the Lovo cell line with si-FOXP1 for 48 h; ^∗^*P* < 0.05, compared with the si-NC and control groups; (d) relative expression of miR-802 after transfection of the Lovo cell line with si-FOXP1 for 48 h; ^∗^*P* < 0.05, compared with the si-NC and control groups.

miR-802 has low expression in gastric cancer, liver cancer, and bladder cancer and has an anticancer effect [14–16]. As one of Fox family members, FOXP1 involved in the development of cardiac myocytes, the differentiation of immune b cells, and the diversity of motor neurons. The expression of Foxp1 was abnormal in many tumors [17–20]. To determine the regulatory effect of overexpressing circIFITM1 on miR-802 and FOXP1, RT-PCR was performed in Lovo cells and displayed that miR-802 was downregulated and Foxp1 was upregulated (Figures 6(a) and 6(b)). After decreasing the expression of circIFITM1 in Lovo cells, miR-802 was upregulated, while FOXP1 was downregulated (Figure 6(c)). After overexpressing miR-802, the expression levels of circIFITM1 and FOXP1 were downregulated (Figures 7(a) and 7(b)). After inhibiting the expression of FOXP1, circIFITM1 expression was downregulated and miR-802 expression was upregulated (Figures 7(c) and 7(d)). These results showed that circIFITM1 suppresses the expression of miR-802, thereby relieving the transcriptional inhibition of miR-802 on its target gene FOXP1.

## 4. Discussion

Circular RNA (circular RNAs) is an endogenous noncoding RNA [21] that regulates gene expression. It can exert an essential regulatory function on gene expression during the transcription or posttranscription processes. The variety of circRNA is very abundant, and it has abnormal expression in colon cancer. It may exert miRNA sponge function to affect the proliferation, apoptosis, and invasion of colorectal cancer, thus participating in pathophysiological processes [22, 23]. HSA displayed a high possibility to be the biomarker of colorectal cancer (CRC) with high sensitivity and specificity [24]. circLMNB1 was highly expressed in tissues and cell lines of CRC, and overexpressing it could suppress the proliferation, migration, and invasion of Lovo cells and promoted cell cycle arrest and apoptosis [25]. Downregulating the circ-BANP expression which was highly expressed in CRC inhibited tumor cell proliferation [26]. In our study, circIFITM1, resistant to actinomycin D treatment, had high stability. The overexpression of circIFITM1 facilitated the proliferative and invasive abilities of Lovo cells, while circIFITM1knockdown suppressed both abilities. The results implied the influence of circIFITM1 on the tumorigenesis and progression of colorectal cancer, pending more evidence to fully confirm in the future. The origins of circIFITM1 are complex, ranging from exons to introns or containing both components [27]. At present, it is believed that the exon-derived circRNA, which primarily positions at the cytoplasm, usually contains numerous binding loci for miRNA regulating the expression of target genes [28]. circIFITM1 originated from the second exon of IFITM1 gene and was stably expressed in human colorectal cancer cell lines. circIFITM1was highly expressed in colon cancer LOVO cell line. There is interaction between CIRCIFITM1 and miR-802; miR-802 targets the 3′UTR of FOXP1. circIFITM1 downregulated the miR-802 expression and upregulated the FOXP1 expression. Our results showed that circIFITM1 affected the proliferation and invasion of Lovo cells via regulating the miR-802/FOXP1 axis. circ_001569 was proved to sponge miR-145, finally increasing the expression level of miR-145 downstream target E2F5, Bag4, and FNL2 and promoting tumor proliferation and invasion [29]. Has_circ_0000523 can activate the Wnt/B-catenin pathway through acting on miR-31 to facilitate the apoptosis of human CRC cells [30]. circACAP2/has-miR-21-5p/Tima1 signal axis participated in regulating the tumorigenesis and progression of CRC [31].

In the CRC cells, circRNA_100290 and has_circ_136666 had been proved to facilitate the proliferation via targeting miR-516b and miR-136/SH2B1, respectively [32, 33].

To sum up, this work firstly showed the increased circIFITM1 level in clinical tissues and cell lines of colon cancer and proved that regulating the circIFITM1 expression could affect the proliferative and invasive abilities of Lovo cells directly via the circIFITM1/mir-802/FOXP1 axis. Henceforth, the role and mechanism of circIFITM1 in colon cancer will be further investigated using animal solid tumor models.

## Figures and Tables

**Table 1:** Primers for gene.

Gene	Primer(5′-3′)
circIFITM1	F: CCTGCCCCTAGATACAGCAG R: GAGACCTCCTTTCCCCTGTC
IFITM1	F:ACTCAACACTTCCTTCCCCAA R:CTTCCTGTCCCTAGACTTCACG
miR-802	F: ACAAGGATGAATCTTTGTTACTG R: TTCACAATGCGTTATCGGATGT
FOX1P	F: CTCAAGGCATGATTCCAACA R: GCATGTGGGTTCATTATTAAGGA
b-Actin	F-GTGGCCGAGGACTTTGATTG R: CCTGTAACAACGCATCTCATATT
U6	F: ATTGGAACGATACAGAGAAGATT R: GGAACGCTTCACGAATTTG
RT-miRNA	CTCAACTGGTGTCGTGGAGTCGGCAATTCAGTTGAGTTCCCAT

## Data Availability

The data used to support the research are included within this article.

## Abbreviations

circRNA: Circular RNA
IFITM1: Interferon-induced transmembrane protein 1
NC: Negative control
RIP: RNA-binding protein immunoprecipitation
RT: Reverse transcription
siRNA: Small interfering RNA
UTR: Untranslated region
FOXP1: Forkhead box p1.
